# HIV-Associated Apathy/Depression and Neurocognitive Impairments Reflect Persistent Dopamine Deficits

**DOI:** 10.3390/cells10082158

**Published:** 2021-08-21

**Authors:** Kristen A. McLaurin, Michael Harris, Victor Madormo, Steven B. Harrod, Charles F. Mactutus, Rosemarie M. Booze

**Affiliations:** Department of Psychology, University of South Carolina, Columbia, SC 29208, USA; mclaurik@email.sc.edu (K.A.M.); mwh2@email.sc.edu (M.H.); vmadormo@email.sc.edu (V.M.); harrods@mailbox.sc.edu (S.B.H.); mactutus@mailbox.sc.edu (C.F.M.)

**Keywords:** dopamine, HIV-1, combination antiretroviral therapy, pre-pulse inhibition, attention, apathy, microglia, dendritic spines

## Abstract

Individuals living with human immunodeficiency virus type 1 (HIV-1) are often plagued by debilitating neurocognitive impairments and affective alterations;the pathophysiology underlying these deficits likely includes dopaminergic system dysfunction. The present review utilized four interrelated aims to critically examine the evidence for dopaminergic alterations following HIV-1 viral protein exposure. First, basal dopamine (DA) values are dependent upon both brain region andexperimental approach (i.e., high-performance liquid chromatography, microdialysis or fast-scan cyclic voltammetry). Second, neurochemical measurements overwhelmingly support decreased DA concentrations following chronic HIV-1 viral protein exposure. Neurocognitive impairments, including alterations in pre-attentive processes and attention, as well as apathetic behaviors, provide an additional line of evidence for dopaminergic deficits in HIV-1. Third, to date, there is no compelling evidence that combination antiretroviral therapy (cART), the primary treatment regimen for HIV-1 seropositive individuals, has any direct pharmacological action on the dopaminergic system. Fourth, the infection of microglia by HIV-1 viral proteins may mechanistically underlie the dopamine deficit observed following chronic HIV-1 viral protein exposure. An inclusive and critical evaluation of the literature, therefore, supports the fundamental conclusion that long-term HIV-1 viral protein exposure leads to a decreased dopaminergic state, which continues to persist despite the advent of cART. Thus, effective treatment of HIV-1-associated apathy/depression and neurocognitive impairments must focus on strategies for rectifying decreases in dopamine function.

## 1. Introduction 

Since the beginning of the acquired immunodeficiency syndrome (AIDS) epidemic, neurocognitive impairments (NCI) and affective alterations have been associated with the disease [1,2]. Early in the AIDS epidemic, underlying focal processes and opportunistic infections accounted for approximately 30% of the neurological complications in individuals with AIDS; a progressive dementia, however, was more commonly reported [3]. The identification of human immunodeficiency virus type 1 (HIV-1) as the retroviral etiology of AIDS [4,5] led to the hypothesis that NCI and affective alterations may result from the direct effect of the virus on the brain. Indeed, HIV-1 penetrates the central nervous system (CNS) early in the course of infection [6], evidenced by the presence of HIV-1 in postmortem brain tissue [7,8,9], findings which led to the characterization of this progressive dementia, which became known as AIDS dementia complex (ADC, also recognized as HIV-associated dementia (HAD)).

ADC, which afflicted approximately 66% of autopsy-verified AIDS patients early in the epidemic, was a neurological syndrome primarily occurring during the later phases of systemic AIDS [3]. Early clinical characteristics of ADC included NCI (e.g., forgetfulness, loss of concentration), affective alterations (e.g., apathy) and motor system deficits [3,10,11]. Across time, most patients with ADC exhibited a steady decline in neurocognitive function, leading to severe dementia, ataxia and motor weakness [10].

Pathologically, distinct abnormalities in the white matter and subcortical structures, including the basal ganglia, were observed in the brains of individuals with ADC [12], observations which led researchers to hypothesize dopaminergic system dysfunction as a potential mechanism underlying the disease [13]. Cerebrospinal fluid (CSF) levels of dopamine (DA [14,15]) and homovanillic acid (HVA [15,16,17]), the primary DA metabolite, were significantly reduced in HIV-1/AIDS patients relative to seronegative controls. In HIV-1-infected brains, significant reductions in tyrosine hydroxylase (TH), the rate-limiting enzyme of DA synthesis, were also observed [18]. Most critically, the relationship between CSF HVA levels and neuropsychological function in HIV-1-infected patients provided compelling evidence for the role of dopaminergic system dysfunction in the pathogenesis of ADC [17].

With the discovery and introduction of antiretroviral therapies, however, AIDS/HIV-1 became a chronic, manageable disease, albeit NCI and affective alterations persist. The development of zidovudine (azidothymidine [19]), the first generation of antiretroviral therapy, provided early evidence that effective inhibition of HIV-1 may have some effects on cognitive function in AIDS patients [20,21,22]. Zidovudine monotherapy did not, however, mitigate affective alterations [20]. The subsequent utilization of multiple antiretroviral compounds to treat HIV-1 (i.e., combination antiretroviral therapy (cART)) led to a dramatic decrease in the severity of NCI and affective alterations associated with HIV-1 [23]. Specifically, in the post-cART era, ADC is rare, afflicting only 2–8% of cART-treated HIV-1 seropositive individuals [23]. However, milder forms of NCI and affective alterations persist, afflicting between 30% and 70% of HIV-1 seropositive individuals [24,25,26,27].

Although the pathophysiology of HAND and affective alterations in the post-cART era is likely multidimensional, dopaminergic system dysfunction persists [28,29,30]. Using four interrelated aims, the present review will examine evidence for alterations in dopaminergic levels in HIV-1 in the post-cART era. Given that approximately 73% of HIV-1 seropositive individuals are currently accessing antiretroviral treatment [31], the present review focuses on studies using biological systems (i.e., HIV-1 seropositive individuals, primates, rats, mice) with viral suppression. First, we will report basal/tonic values of DA in the CNS, including a discussion of the experimental approaches (e.g., high-performance liquid chromatography (HPLC), microdialysis, fast-scan cyclic voltammetry (FSCV)) used to measure DA. Second, the present review will examine the prominent evidence, including both anatomical and clinical symptomology, for the persistent decreased dopamine in HIV-1 seropositive individuals. Third, the potential effects of cART on the dopaminergic system will be assessed. Finally, we will address the mechanistic implications for dopamine decreases in HAND.

## 2. Basal Dopamine Concentrations in the Central Nervous System

The physiological significance of DA [32], and its presence in the brain [33,34,35], was first established in the 1950s. Subsequent methodological advances, including the development of microdialysis, HPLC and FSCV, afforded a critical opportunity to detect changes in basal (or tonic) DA [36]. However, DAs precise influence on cognition and behavior remains unclear, in large part due to inconsistencies in measured DA levels. Thus, one of the primary goals was to illustrate the inconsistencies in values via examination of the standard error of the mean and relative standard error.

DA concentration was estimated using the reported means, which were converted into ng/g of tissue (Table 1). Reported estimates are collapsed across species and biological sex under the assumption that the variability between brain regions and methodological approach are greater than the variability between species and sex [37]. Each manuscript, therefore, provided a single observation for each brain region that was reported. All estimates, as well as information regarding species and biological sex, are reported in Supplementary Table S1.

**Figure 1:**
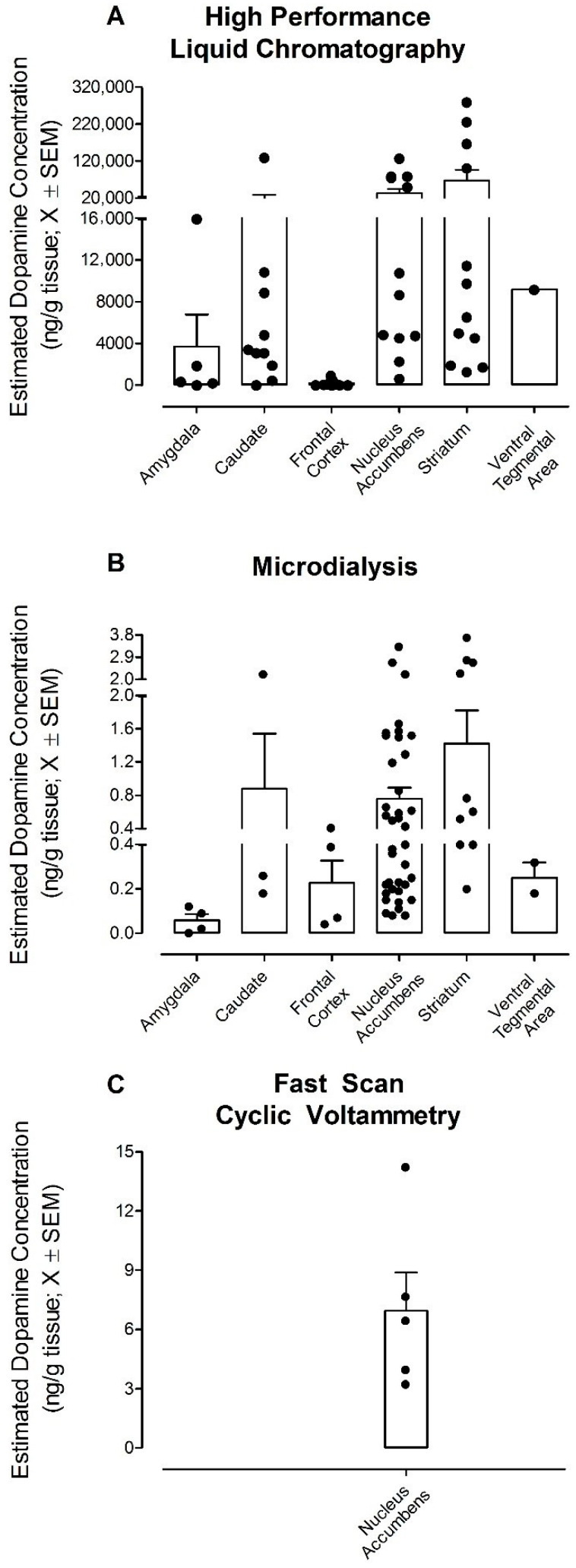
Graphical illustration of the profound differences in estimated dopamine (DA) concentration (ng/g of tissue; X ± SEM) dependent upon methodology (i.e., (**A**) high-performance liquid chromatography, (**B**) microdialysis, (**C**) fast-scan cyclic voltammetry) and brain region. Each dot represents the estimated DA concentration from a study.

Critical evaluation of the literature revealed that basal DA values are dependent upon not only brain region, but also methodological technique (between-subjects ANOVA with log estimated DA concentration in ng/g of tissue as the dependent variable: brain region by method interaction, *F*(5,104) = 7.05, *p* ≤ 0.001, η_p_^2^ = 0.253; Table 1; Figure 1). For example, utilization of HPLC to measure DA in the nucleus accumbens (NAc) results in an average estimated DA concentration over 40,000 times greater than the average estimated DA concentration measured using microdialysis. This outcome might be anticipated due to tissue homogenization prior to HPLC measurement; HPLC, therefore, measures total tissue DA content, whereas microdialysis measures extracellular DA levels [38]. Additionally, substantial variability in reported basal DA values within a single methodological approach was observed. For example, the relative standard error for the NAc was 33.9%, 17.1% and 27.8% for HPLC, microdialysis and FSCV respectively, values which are even higher in other brain regions (e.g., amygdala: 84.1% (HPLC) and 50% (microdialysis)). Given the substantial variability within and between methodological techniques, a brief discussion of some of the critical experimental considerations underlying these discrepancies is warranted. In addition, the potential utility of the latest technology (i.e., G protein-coupled receptor (GPCR) biosensors) for monitoring DA release is briefly reviewed.

### 2.1. High-Performance Liquid Chromatography (HPLC)

Broadly, chromatography is a well-established separative and analytical technique introduced by James and Martin [115]; the emergence of HPLC, however, is attributed to Huber and Hulsman [116]. To conduct HPLC, a pressurized liquid solvent (i.e., mobile phase) containing the sample is passed through a column filled with a solid adsorbent material, and each compound elutes at a unique rate, resulting in the separation of components as they flow through the column [117]. The isolated compounds are subsequently identified and quantified using a detector (e.g., UV/Vis spectrometry). HPLC can be further subdivided into multiple types dependent upon the type of column (e.g., liquid–liquid, ion-exchange, size exclusion) and “mobile phase” (e.g., non-polar, polar), factors which influence sensitivity, resolution and the method of brain tissue extraction. Critically, differences in extraction methodology (e.g., time of initial extraction, aqueous pH value, extraction solvents) result in drastic differences in percent recovery, an indirect measure of basal DA concentration [118].

Given HPLC’s wide use, sources of random and systematic error in HPLC have been studied extensively [119]. The brief discussion in the present review will focus on sources of error reported to affect the electrochemical detection of DA. First, the mobile phase column composition (e.g., ion pairing agent type, organic modifier, pH) has a pronounced effect on the capacity factor (k’), retention time, peak height units of DA and peak symmetry [120,121,122,123,124]. Second, the flow gradient rate, similarly, has a prominent effect on the resolution of the eluting compounds, k’ and background current [125]. Finally, chromatographic instrumentation, including the column temperature, alters the retention time of DA, whereby an increase in column temperature is associated with a decrease in retention time [123]. Additionally, column age may influence the resolution between DA and its metabolite (i.e., 3,4-Dihydroxyphenylacetic acid), whereby decreased resolution has been observed after approximately 500 injections of the biological material directly onto the column top [121]. 

### 2.2. Microdialysis

**Figure 2:**
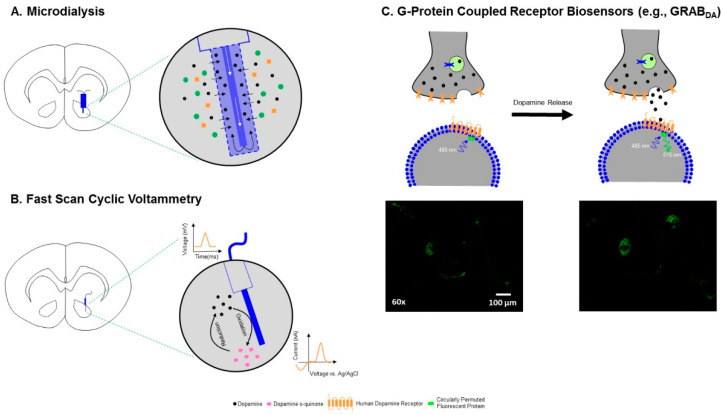
Technical illustration of three of the prominent methods utilized to detect dopamine (DA) levels in the CNS. Given that high-performance liquid chromatography (HPLC) is more classically used for analyte quantification on brain tissue homogenates or following microdialysis, the method is not illustrated. (**A**) During microdialysis, a probe composed of a semipermeable dialysis membrane is surgically implanted into the brain, and a perfusion medium (white arrows) is infused slowly and continuously. During perfusion, molecules in the extracellular space diffuse through the semipermeable membrane, and are transported into outflow tubing and collected for analyte quantification (e.g., HPLC). (**B**) In fast-scan cyclic voltammetry, a small carbon-fiber microelectrode is surgically implanted into the brain. The voltage potential at the carbon-fiber microelectrode is rapidly increased and decreased, resulting in the oxidation and reduction of DA. During the oxidation and reduction processes, the transfer of electrons is measured in current at the surface of the carbon-fiber microelectrode, and the amount of current can be subsequently converted into the concentration of DA. Additionally, the voltammogram is used for analyte identification, whereby DA exhibits one oxidation and one reduction peak. (**C**) More recently, G-protein coupled receptor (GPCR) biosensors for DA have been developed, affording an opportunity to track the release dynamics of DA. DA biosensors have a circularly permuted fluorescent protein (e.g., Green: cpGFP, Red: cpmApple) inserted into the third intracellular loop of the DA receptor. When DA binds to the endogenous ligand, the GPCR exhibits a conformational change, resulting in an increased fluorescent intensity. Our laboratory has recently transfected cells with GRAB-DA2m, a DA receptor 2 subtype biosensor, in vitro. Upon stimulation with 100 nm DA, an increase in the fluorescence intensity of cpGFP is observed.

The utilization of microdialysis to quantify neurotransmitters in the brain was first reported in the 1970s and 1980s [126,127,128], research which contributed significantly to the widespread implementation of microdialysis methods. Microdialysis relies on the principle of diffusion, whereby molecules move from an area of high concentration to an area of low concentration. Methodologically, a microdialysis probe composed of a semipermeable dialysis membrane is surgically implanted into the brain, and a perfusion medium is infused slowly and continuously [129]. During perfusion, molecules in the extracellular space diffuse through the semipermeable membrane, are transported into outflow tubing and are collected for analyte quantification (e.g., HPLC [129]; Figure 2A). Although microdialysis detects neurotransmitters at low- to sub-nanomolar levels (for DA, see [113]), the technique has relatively low spatiotemporal resolution and is unable to evaluate real-time changes in the neurochemical environment.

Despite being considered the “gold standard” for obtaining basal neurotransmitter levels, methodological limitations may impede precise and/or consistent measurements. The diameter of a typical microdialysis probe is approximately 300 μm, a size which is substantially larger than neurons and glial cells (5–100 μm), as well as blood capillaries (8–10 μm) and vessels (~1 mm) in the brain [130]. Implantation of microdialysis probes, therefore, damages brain tissue, as evidenced by signs of ischemia [131,132] and a compromised blood–brain barrier [131,133]. Additionally, tissue damage resulting from the microdialysis probe disrupts synapses and neurons [134]. Critically, dopaminergic activity is disrupted by the implantation of microdialysis probes, as evidenced by both decreased DA release over post-probe implantation time [135] and alterations in the amplitude of evoked responses [136,137]. Recently developed novel approaches, including pharmacological agents [138,139] and a microfabricated probe [140], have the potential to mitigate some of the concerns regarding tissue disruption. 

Consistent measurement of basal DA levels is further dependent upon multiple methodological details. Although HPLC is often used as a method to quantify the output from microdialysis, the methodological details discussed within the present section are conducted prior to the quantification of analytes. First, inappropriate concentrations of specific ions (e.g., Ca^2+^, NA^+^, K^+^) in the perfusate medium disrupt the homeostatic balance of the extracellular environment, altering the basal DA concentration. For example, increases in basal DA concentration are observed when the perfusate medium contains higher (e.g., 3.4 mM) levels of Ca^2+^ [63,141] or K^+^ [128]. In sharp contrast, utilization of a perfusion solution with too little Ca^2+^ [128,142] or too little K^+^ [142] results in decreased extracellular DA levels. It is vital, therefore, that the composition of perfusion solutions mimic the brain extracellular fluid; additional parameters, including pH and temperature, are also critical considerations [143]. Second, substantial increases in the concentrations of extracellular DA occur immediately following death [144,145,146]. Basal DA levels subsequently decrease as the postmortem interval increases [144,145,146]; albeit, basal DA concentration remains elevated, relative to pre-death levels, for at least an hour postmortem [144,145]. Third, in neutral and basic aqueous solutions, DA degrades rapidly [147], including in many common (e.g., aCSF, brain dialysate) perfusion solutions [148]. Several approaches, including temporal proximity (i.e., minimization of the time between sample collection and analysis [149]), addition of stabilizing agents to either the collection bins [65] or microdialysis media [150] and a microdialysis/LCMS system [148], have been implemented to mitigate the DA instability problem. Despite the validity of these approaches, inter-laboratory differences may preclude determining an estimate of the “true” basal DA concentration.

### 2.3. Fast-Scan Cyclic Voltammetry (FSCV)

FSCV, an electroanalytical technique developed in the early 1980s [151,152], affords a method to detect rapid neurotransmitter dynamics in the brain [153]. From a theoretical perspective, FSCV relies upon chemical sensing of neurotransmitters at carbon-fiber microelectrodes. Specifically, the voltage potential at the carbon-fiber microelectrode is rapidly increased and decreased, resulting in the oxidation and reduction of electroactive substances [154]. Examination of the cyclic voltammogram, which presents data as time (*x*-axis) by voltage (*y*-axis), allows for compound identification [155,156]. The strengths of FSCV include its high spatial (micrometer) acuity, high temporal (sub-second) resolution and high chemical (nanomolar range) sensitivity. However, FSCV is limited by the need for digital background subtraction [157], which restricts measurements to relative neurotransmitter changes, a factor which precludes the measurement of basal concentrations of electroactive species [154]. Therefore, FSCV has typically been utilized to measure phasic, rather than tonic, DA release. Recent novel modifications have afforded an opportunity to investigate tonic DA concentrations using FSCV [103,104,158,159]. While an in-depth discussion of these modifications is beyond the scope of this review, it is an emerging area of research that has the potential to transform our ability to accurately measure basal DA levels.

### 2.4. G Protein-Coupled Receptor (GPCR) Biosensors

GPCR biosensors for DA (or DA biosensors), the most recent method developed for monitoring DA dynamics, were first reported in 2018 [160,161], and contemporary versions have expanded upon these initial reports [162,163]. Theoretically, fluorescent DA biosensors rely upon the interaction between DA and D_1_- and D_2_-like GPCRs. DA biosensors were developed by inserting a genetically encoded, circularly permuted fluorescent protein (e.g., Green: cpGFP, Red: cpmApple) into the third intracellular loop of the naturally occurring human DA receptor. When DA is released, it binds to the endogenous ligand, causing a rapid conformational change in the GPCR, a conformational change that induces a profound increase in fluorescence intensity (i.e., 90–900%, for a review, see [164]; Figure 2C). DA biosensors exhibit high selectivity, molecular specificity, affinity (sub-micromolar) and resolution (sub-second [160,161,162,163,165]), making them ideally suited for tracking DA release. However, DA biosensors may be limited by low basal fluorescence levels, which precludes the detection of basal DA levels. A more comprehensive discussion of GPCR biosensors for DA is provided by Labouesse et al. [164].

### 2.5. General Experimental Considerations

Ideally, an estimate of basal DA values would be highly replicable when measurements are obtained in the same brain region, using the same methodological technique and in nearly genetically identical animals. However, basal DA concentrations are altered by natural biological variation within and between subjects. Independent of species, there is natural biological variation in basal DA concentrations resulting from within and between subject’s factors. For example, basal extracellular DA levels change across the functional lifespan, with significantly decreased DA observed in aged, relative to young, animals [166]. Furthermore, basal DA levels in the NAc [167,168], striatum [149,169] and medial prefrontal cortex (mPFC [170]) fluctuate in a circadian rhythm. Additionally, hormones have a profound impact on basal DA levels, as evidenced by changes across the estrous cycle [171,172] and resulting from gonadectomy [173].

To date, the substantial variability between studies, even within a single methodological approach, has obfuscated our ability to experimentally determine the “true” basal DA concentration. When appropriate experimental controls are implemented, the impact of a treatment (e.g., HIV-1, substance use) on basal DA concentration can be reliably determined; comparing between studies, however, remains challenging. Stringent and detailed reporting of methodological procedures may aid in determining which studies can be most accurately compared. From a practical perspective, however, the information compiled in Table 1 (expanded in Supplementary Table S1) provides a summary of the techniques currently in use.

In sum, HPLC of tissue homogenates may reveal total DA tissue content, while microdialysis enables sampling of the extracellular basal DA levels, but lacks temporal resolution (minutes) and spatial resolution. FSCV is currently used for relative changes in DA signals, and not for assessing basal DA levels. Although GPCR biosensors for DA may not clarify basal DA levels in the brain, their ability to rapidly detect DA function has the potential to transform our understanding of neural circuits. Critically, each neurochemical method for assessing DA levels has benefits and limitations that must be weighed when designing an experiment.

## 3. Chronic HIV-1 Results in Decreased Dopamine

Inconsistencies in the estimated basal DA values does not preclude the utilization or importance of these methodological techniques for evaluating group differences. As demonstrated in Table 2, HPLC, microdialysis and FSCV have been fundamental in elucidating how HIV-1 viral protein exposure alters basal DA concentration relative to seronegative individuals or controls. Results (Table 2) overwhelmingly support decreased DA concentrations following chronic HIV-1 viral protein exposure in either HIV-1 seropositive humans or biological systems utilized to model HIV-1.

Despite the overwhelming support for a hypodopaminergic state in HIV-1 (i.e., low levels of DA), there are a few outliers. Three studies [174,175,176] have reported transitory increases in DA concentration in the CSF, caudate putamen and prefrontal cortex (PFC), respectively. The subjects (i.e., humans, mice) evaluated in these studies share a key commonality: early or acute HIV-1. Specifically, the clinical sample included individuals in clinical stage 1 [174], which is characterized by asymptomatic infection and persistent generalized lymphadenopathy [177]. Preclinical measurements were conducted either one [176] or three [175] days after the completion of Tat protein induction by a doxycycline regimen. Critically, these increases in DA either failed to persist for longer intervals after Tat protein induction (i.e., 10 Days: [178], 40 Days: [175]) or were brain region-specific [176]. Moreover, there is no evidence for a hyperdopaminergic state during chronic HIV-1 infection in humans, suggesting little clinical relevance for assessing acute increases in dopamine.

Another notable inference that can be drawn from Table 2 regards the influence of cART on DA function in HIV-1 seropositive individuals. While monotherapy, including zidovudine (azidothymidine), was first implemented in 1985 [19], cART began in 1996. Critically, the strong support for decreased dopaminergic function spans across studies in both the pre- and post-cART era. A more comprehensive discussion for the potential role of cART in dopaminergic system dysfunction is presented in Section 5.

Undoubtedly, long-term HIV-1 viral protein exposure leads to persistent DA deficits, independent of treatment with cART. It is possible that there is an initial transient increase in DA immediately following HIV-1 infection, given the increased life expectancy for HIV-1 seropositive individuals [179,180], however, the acute phase fails to accurately reflect the current clinical syndrome.

## 4. HIV-1 Clinical Symptoms Reflect a Hypodopaminergic State

In 2007, the nosology for neurological complications in HIV-1 seropositive individuals was updated to reflect the milder phenotype of NCI and affective alterations, collectively termed HIV-1-associated neurocognitive disorders (HAND), observed in the post-cART era [190]. Using the established criteria, HIV-1 seropositive individuals are classified into one of three categories (i.e., asymptomatic neurocognitive impairment (ANI), mild neurocognitive disorders (MND) or HAD) based on neurocognitive performance and alterations in daily functioning [190]. HAND, a progressive disease [191,192,193,194,195], is characterized by prominent neurocognitive deficits in speed of information processing, attention, working memory and executive function [26,196,197]. Affective alterations commonly observed in HAND include apathy [27,198] and depression [199,200]. These clinical symptoms reflect persistent DA deficits in HIV-1 seropositive individuals.

**Figure 3:**
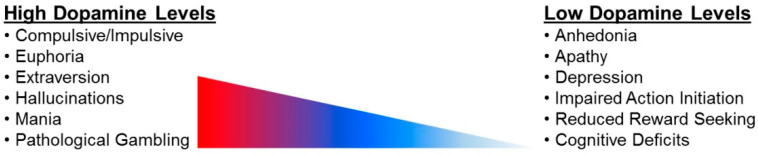
Overview of behaviors characteristic of a hyperdopaminergic versus hypodopaminergic state.

The present review will focus on evaluating the role of DA in the regulation of pre-attentive processes, attention and apathy, as a discussion of all neurocognitive and/or affective alterations, neural circuits and/or cellular mechanisms is beyond the scope. However, it is notable that the effect of decreased DA availability in HIV-1 seropositive individuals extends more broadly, as it is significantly associated with neuropsychological performance [29] and depression [188]. Furthermore, Figure 3 illustrates the profound difference in the clinical symptoms of hyperdopaminergic versus hypodopaminergic systems. There is no clinical evidence supportive of high dopamine levels following chronic HIV-1 infection in humans, suggesting that models/therapeutics must focus on rectifying low dopamine levels.

### 4.1. Pre-Attentive Processes

Pre-attentive processing (or sensorimotor gating) is defined as the screening of extraneous information to facilitate the uninterrupted processing of relevant information. Pre-pulse inhibition (PPI) of the auditory startle response (ASR), popularized by Hoffman and Ison [201,202], affords a translational experimental paradigm to measure pre-attentive processes. The presentation of a discrete pre-stimulus prior to a startling stimulus attenuates an individual’s startle response during a brief temporal window (i.e., 30–500 msec [203]). Prominent impairments in PPI have been observed in multiple neuropsychological disorders, including schizophrenia [204], obsessive-compulsive disorder [205,206], Huntington’s disease [207] and HIV-1 [208,209]. In HIV-1, deficits in PPI are characterized by a reduction in percent PPI [208,210] and a relative insensitivity to the manipulation of interstimulus interval (ISI, i.e., time between the discrete pre-stimulus and startling stimulus [209,211]). Most critically, impairments in PPI resulting from chronic HIV-1 viral protein exposure are associated with alterations in higher-order cognitive processing [208], progress across the functional lifespan [212,213] and may serve as a diagnostic and/or prognostic biomarker for HAND [214].

PPI is regulated, at least in part, by brain regions integral to the fronto-striatal circuit (i.e., ventral tegmental area (VTA), NAc and PFC) and the dopaminergic system. Specifically, within the neural circuit mediating PPI, the NAc is innervated by dopaminergic projections from the VTA and glutamatergic afferents from the mPFC. Gamma aminobutyric acid (GABA) projections are subsequently relayed from the NAc to the pedunculopontine tegmental nucleus (PPTg). Information is then sent from the PPTg to the caudal pontine reticular nucleus, a component of the acoustic startle circuit [215], resulting in the elicitation of a startle response. The auditory startle pathway and entire neural circuitry underlying PPI is more comprehensively reviewed by Koch [216] and Fendt et al. [217].

Profound reductions in PPI are observed when pharmacological manipulations and lesioning approaches are utilized to induce a hypodopaminergic state. Apomorphine, a direct dopamine agonist, acts in a biphasic dose-dependent manner [218,219], whereby low doses act on presynaptic receptors, resulting in decreased dopaminergic tone; high doses of apomorphine, in sharp contrast, act on both pre- and post-synaptic receptors, resulting in a hyperdopaminergic state. Administration of low doses of apomorphine, independent of sensory modality (i.e., auditory, visual [220]) or ISI [221], leads to prominent reductions in PPI [220,221,222]. Selective D1 receptor antagonists, including SCH23390 [223,224] and SCH39166 [225], also reduce PPI when injected into either the PFC [223,225] or dorsal striatum [224]. Furthermore, inducing a hypodopaminergic tone via 6-hydroxydopamine (6-OHDA) injections, which destroys dopaminergic and noradrenergic neurons [226,227], reduces PPI [228,229]. Collectively, pre-attentive processes, as indexed by PPI, are dramatically reduced under conditions that mimic a hypodopaminergic state, reductions which are similar to those observed in HIV-1 in the post-cART era.

### 4.2. Attention

Attention is a biologically complex cognitive function dependent upon reciprocal excitatory and inhibitory processes [230]. By definition, attention is selective, requiring the brain to process the most relevant information, while excluding, or inhibiting, irrelevant information [230]. According to a hierarchical model proposed by Sohlberg and Mateer [231,232], attention can be divided into multiple subcomponents (i.e., arousal, focused attention, sustained attention, selective attention, alternating attention and divided attention). With regards to HIV-1, chronic HIV-1 viral protein exposure induces prominent deficits across the subcomponents of attention, including sustained attention or vigilance [233], selective attention [234,235] and divided attention [236].

Although the precise neural circuitry underlying attentional processes has not yet been fully elucidated, there is strong evidence for the fundamental role of the PFC in higher-order cognition. The PFC is divided into six layers, superficial to deep, and is comprised of three major subdivisions, including the lateral PFC (lPFC), mPFC and orbital PFC (oPFC [230]). Midbrain DA neurons project to the PFC via the mesocortical DA pathway, a pathway which can be divided into two parallel systems [237]. Specifically, DA afferents from the VTA innervate the mPFC, whereas the lPFC is innervated by DA projections from the substantia nigra [237]. Most critically, however, DA modulates cognitive processes, including attention, in the PFC.

Induction of a hypodopaminergic state via pharmacological manipulations or lesioning approaches disrupts attentional behavior. First, local administration of the selective D1 antagonist SCH23390 to either the PFC [238,239] or NAc [240] impairs attention. Infusion of the D2 receptor antagonist sulpiride into the NAc [240], but not the PFC [238], also decreased attentional accuracy. Second, neonatal treatment with 6-OHDA produces persistent marked impairments in selective, spatial and/or sustained attention at a juvenile stage [241,242], during adolescence [243] and in adulthood [244]. 6-OHDA lesions of the PFC during adulthood also reduced selective attention, as evidenced by an increased susceptibility to task-irrelevant distractors [245]; attentional set shift, however, is relatively spared [245,246], consistent with observations following chronic HIV-1 viral protein exposure [233]. Furthermore, chronic administration of the selective dopaminergic neurotoxin 1-methyl-4-phenyl-1,2,3,6-tetrahydropyridine (MPTP) induced attentional deficits, characterized by impairments in sustained spatial attention and focused attention [247]. Taken together, induction of a hypodopaminergic state produces marked impairments in attentional processes similar to those observed in HIV-1 in the post-cART era.

### 4.3. Apathy

Traditionally, apathy has been defined as a lack of motivation [248] that is evidenced by the quantitative reduction in voluntary and goal-directed behaviors [249]. Goal-directed behaviors require the use of action to translate an internal state into the attainment of a goal. In clinical studies, apathy is most commonly [250] assessed using either the Apathy Evaluation Scale [251] or the Neuropsychiatric Inventory [252], scales which exhibit both strong reliability and validity [250]. Furthermore, preclinical studies have utilized operant and Pavlovian conditioning as a method to evaluate how willing an animal is to “work” for reinforcement [198,253,254]. Understanding apathy from both a clinical and preclinical perspective is vital, given its prevalence in many neurological disorders (e.g., Alzheimer’s disease [255], Parkinson’s disease [256], HIV-1 [27,257]). Indeed, chronic HIV-1 viral protein exposure induces prominent alterations in goal-directed behaviors [198,254]. The clinical significance of apathy in HIV-1 seropositive individuals cannot be understated, as increased apathy is significantly associated with greater impairments in activities of daily living [27,258], decreased medication adherence [259] and decreased quality of life [260].

Apathy is regulated, at least in part, by the anterior cingulate circuit, one of the behaviorally relevant fronto-striatal circuits [261]. Within this circuit, projections from the anterior cingulate cortex innervate the ventral striatum, including the NAc [262]. Subsequently, neurons in the ventral striatum project to the globus pallidus interna, ventral pallidum and rostrodorsal substantia nigra [263]. Both the ventral striatum and anterior cingulate cortex receive dopaminergic innervation from the VTA, supporting the fundamental role of DA in apathetic behaviors.

Indeed, the reduction of dopaminergic signaling via lesioning and chemogenetic approaches have demonstrated the importance of the neurotransmitter in goal-directed behavior. Induction of hypodopaminergic tone via either 6-OHDA lesions of the substantia nigra pars compacta [264,265,266] or MPTP [267] impaired motivated behaviors. More recently, the chemogenetic inhibition of DA neurons in the VTA dose-dependently reduced effort-based motivation [268]. Collectively, strong evidence supports apathetic behaviors under hypodopaminergic states.

## 5. Role of cART in Dopaminergic System Dysfunction

Currently, approximately 30 antiviral drugs are approved for the treatment of HIV-1 [269]. The approach to HIV-1 treatment evolved from the use of monotherapy with the nucleoside reverse transcriptase inhibitor (NRTI) zivodudine, to various combinations of two to four compounds composed of a NRTI, integrase strand inhibitor (INSTI), protease inhibitor (PI), or non-nucleoside reverse transcriptase inhibitors (NNRTI). Given that a hypodopaminergic tone is observed following chronic HIV-1 viral protein exposure, it is vital to examine the potential role of cART in dopaminergic dysregulation.

Some cART drugs, particularly those with greater CNS penetrance [270], are associated with adverse psychoactive effects in HIV-1 seropositive individuals [271,272,273,274]. Specifically, NRTIs, including efavirenz, are most commonly associated with adverse neuropsychiatric outcomes [275]. Patients commonly report hallucinations, delusion, paranoia and mania, as well as depression, anxiety, nervousness, dizziness, sleep disturbances and abnormal dreams [271,272,273,274].

Efavirenz exhibits a complex neuropharmacological profile, whereby it interacts with serotonin (5-HT) and GABA_A_ receptors, and multiple monoamine transporters (i.e., serotonin transporter (SERT), dopamine transporter (DAT), vesicular monoamine transporter 2 (VMAT2) [276,277,278]). Further evidence for these interactions is provided by increased basal levels of 5-HT, DA, and norepinephrine, albeit in a region-specific manner, following intraperitoneal injections of 5 mg/kg of efavirenz every other day for two weeks [279]. Under differing experimental conditions, acute, oral administration of efavirenz (0, 25, 50 mg/kg) dose-dependently increased striatal DA levels; however, no significant alterations in basal DA levels were observed after sub-chronic (i.e., two-week) exposure [280]. Highly translational behavioral procedures examining a drug’s pharmacodynamic activity (e.g., drug discrimination, sensitization/habituation) and DA-related behaviors (e.g., drug reinforcement, conditioned reward behaviors) support the observed neuropharmacological profile and will be discussed in turn below.

### 5.1. Drug Discrimination

Drug discrimination is a free-operant procedure that allows the animal to learn that a subjective drug effect (i.e., discriminative stimulus) sets the occasion for reinforcement of a particular response [281]. Specifically, following injection of a psychoactive drug, responses on one levers are reinforced. Whereas, following a saline, however, responses on the alternative lever are reinforced [282]. After the discrimination is learned, a novel drug can be injected to determine whether the training and test drug share discriminative stimulus properties. If the novel test drug produces a discriminative stimulus similar to the training drug, then animals will respond on the lever associated with the training drug; however, the rat will respond on the saline-associated lever if the novel drug discriminative stimulus is different than that of the training drug.

Using the drug discrimination procedure, Gatch et al. [276] examined whether efavirenz produced a discriminative stimulus similar to different drugs of abuse. First, a group of rats were trained to discriminate the subjective drug effect of lysergic acid diethylamine (LSD), a serotonin receptor agonist, from that produced by saline injection. When tested with various doses of efavirenz, rats responded as if LSD was onboard, an effect that is primarily mediated by the 5-HT_2A_ receptor. Results were confirmed by training a separate group of rats to discriminate an efavirenz-induced drug state from saline. Under testing conditions with LSD, animals responded on the lever associated with the training drug (i.e., efavirenz) rather than the saline-associated lever. Second, a separate group of rats were trained to discriminate the subjective drug effect of cocaine, a DA reuptake inhibitor, from saline. When rats were tested with various doses of efavirenz, they primarily responded on the saline-associated lever. Collectively, the pharmacodynamic activity of efavirenz resembles that of the 5-HT receptor agonist LSD.

### 5.2. Sensitization

Repeated exposure to psychoactive stimulants produces prominent behavioral changes (e.g., amphetamine [283], cocaine [284]). Specifically, following acute treatment with psychostimulants, laboratory animals exhibit hyperactivity. Repeated drug exposure, however, induces a progressive and persistent increase in hyperactive behavior, commonly termed “behavioral sensitization”. Critically, the later stages of behavioral sensitization are accompanied by significant elevations in DA in response to a drug [285,286]. Measurement of locomotion following a drug challenge is the classic approach to evaluating behavioral sensitization.

With regards to efavirenz, there is no compelling evidence for behavioral sensitization. Rather, locomotor activity was suppressed in a dose-dependent (3, 10, 30 mg/kg, IP) manner following repeated administration. Critically, the time-course of the efavirenz-induced suppression for the highest dose (30 mg/kg) was nearly identical to that produced by LSD (3 mg/kg [276]). Furthermore, efavirenz increased head-twitching, a behavioral measure commonly utilized to profile serotonergic-like compounds; an increase that was abolished in 5-HT_2A_ receptor knockout mice [276]. More recently, oral efavirenz (0, 25, 50 mg/kg) failed to produce sensitization or suppression of line-crossings in an open-field test [280]. Similarly, Möller et al. [279] observed no statistically significant effect of efavirenz (5 mg/kg of efavirenz every other day for two weeks) on locomotor activity. Thus, the profile of efavirenz is again consistent with a serotonergic pharmacodynamic.

### 5.3. Drug Self-Administration

Preclinical drug self-administration procedures, which evaluate DA-related behaviors, became popularized in the 1960s with the advent of reliable, automated methods for intravenous (IV) drug self-administration [287,288]. The utility of preclinical drug self-administration procedures derives from both their face (i.e., animals self-administer addictive substances commonly abused by humans [198,289,290]) and predictive (i.e., successful identification of substance with high abuse liability; for review, see [291]) validity. Various routes of administration (e.g., oral [290], IV [198]) can be utilized in drug self-administration experimental paradigms to accurately model drug self-administration in humans. For IV drug self-administration, rats are implanted with chronic indwelling jugular catheters and are trained to self-administer drugs by pressing a lever within operant conditioning chambers [287,292]. Similar to other reinforcers, dependence-producing drugs readily maintain behavior on various schedules of reinforcement.

To assess the reinforcing properties of efavirenz, animals were trained to self-administer the DA reuptake inhibitor, cocaine. Following stable self-administration behavior, IV cocaine was replaced with incrementally increasing doses of IV efavirenz (1.0, 0.32, 3.2, or 10.0 mg/kg per infusion). However, independent of dose, lever pressing dramatically decreased during tests in which IV efavirenz was the available reinforcer [276]. Thus, efavirenz fails to maintain operant (goal-directed) behavior [276], indicating that it lacks reinforcing properties associated with drugs that release DA throughout the fronto-striatal system [293].

### 5.4. Conditioned Place Preference

Conditioned place preference (CPP) is a Pavlovian learning procedure that evaluates the rewarding effect of a drug [294]. The CPP procedure repeatedly conditions an animal to two stimuli: a conditional stimulus (CS) and an unconditional stimulus (US). Specifically, one CS (e.g., dark environmental context) is paired with drug treatment (i.e., US). A second CS (e.g., bright environmental context) is paired with no drug treatment. Following conditioning, animals are tested, whereby the rodent can freely move between the drug-paired and non-drug-paired contexts. During testing, a CPP is learned if the animal spends relatively more time in the drug-paired context. Likewise, a conditioned place aversion (CPA) is learned if an animal spends an increased amount of time in the non-drug-paired context. Drugs that produce DA release and maintain self-administration also produce CPP (e.g., cocaine [295]); however, not all drugs that produce CPP are considered dopaminergic drugs [296]. Overall, the CPP experimental paradigm models a Pavlovian conditioning aspect of drug-taking behavior related to incentive salience conditioned to drug-associated stimuli [297].

Mixed findings fail to provide strong support for efavirenz-mediated conditioned reward learning [276,279]. Specifically, under one experimental condition, no dose of efavirenz tested (5–20 mg/kg, IP) produced CPP in rats [276]. On the other hand, dose-dependent changes in behavior, ranging from CPP (5 mg/kg of efavirenz) to CPA (20 mg/kg of efavirenz), were observed [279]. The CPP procedure is not considered an exclusive screen for dopaminergic drugs, as there are serotonin agonists (e.g., LSD, buspirone) that function as an US to produce CPP [294,296,298]. Thus, it is conceivable that the CPP observed by Moller et al. [279] was mediated by efavirenz’s US effects on the serotonergic system [296,298].

### 5.5. Conclusions

Collectively, there is no compelling evidence that efavirenz binds to DA receptors (D1, D4 human; D2, D3 rat) or alters DA reuptake. Efavirenz does inhibit DA reuptake in human cloned DAT [276] and acutely increases basal DA levels [279,280]. However, highly translational behavioral procedures fail to support DA behaviors. Specifically, rats experience different interoceptive cue states when treated with systemic cocaine and efavirenz, as evidenced within a drug discrimination experimental paradigm. Furthermore, efavirenz fails to induce sensitization [276,279,280] and is not self-administered by rats [276]. The hypodopaminergic tone observed following chronic HIV-1 viral protein exposure in both the pre- and post-cART eras (Table 2 above) adds additional credence to these observations. Efavirenz, however, resembles an LSD-like drug consistent with putative effects on the serotonergic system, effects which may produce adverse psychiatric alterations in HIV-1 seropositive individuals [276]. Thus, if cART contributes negatively to HAND, it is unlikely that it results from direct protein interactions to alter dopamine transmission via VMAT2, DAT, or DA receptors.

Whether efavirenz, or other cART-approved drugs, functionally alter DA release throughout fronto-striatal systems is a fundamental question. However, to date, few experiments have directly examined if and/or how cART drugs alter DA function. In addition to the reviewed studies examining efavirenz, the PIs ritonavir and saquinavir failed to alter DA release in rodent hypothalamic tissue [299]. Although the currently available data fail to support the impact of cART on DA, there remains a critical need for additional studies of individual and combinations of cART drugs.

## 6. Mechanistic Implications for Low Dopamine Levels

### 6.1. Homeostatic Conditions

**Figure 4:**
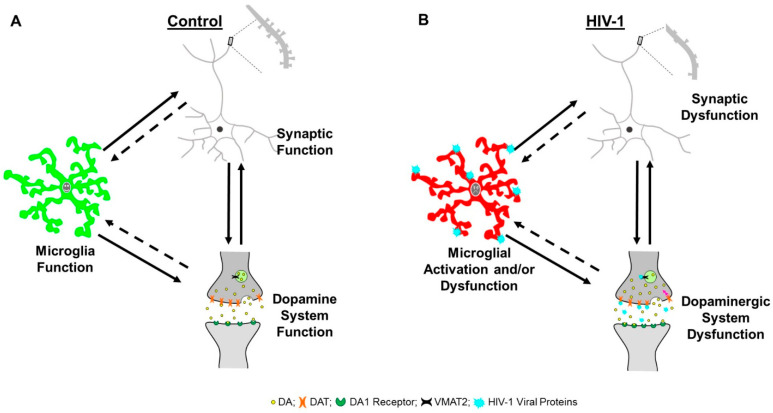
Proposed (potentially) cyclical model of the interrelationship between microglia, and synaptic and dopaminergic system function. (**A**) Under homeostatic conditions, microglia influence both the dopamine (DA) system and synaptic maintenance. Furthermore, synaptic and dopaminergic system functions exhibit a bidirectional relationship. To date, it is unknown whether the relationships between microglia and synaptic function or microglia and DA system function are bidirectional, as indicated via the dashed lines. (**B**) Chronic exposure to HIV-1 viral proteins induces activation, dysfunction and/or senescence of microglia, and microglial alterations which may underlie the prominent low DA levels and/or synaptic dysfunction observed in the post-cART era. DA: dopamine; DAT: dopamine transporter; DA1 Receptor: dopamine 1 receptor; VMAT2: vesicular monoamine transporter 2.

Microglia, which represent 5–20% of adult brain cells [300], belong to the myeloid phagocytic/monocytic lineage [301,302] and serve as resident innate immune cells in the CNS. Morphologically, microglia are characterized by a small soma and slender, highly branched processes [303,304]. In the healthy brain, “resting” microglia utilize their branched processes to continuously survey the environment [303,304]; environmental surveillance which is uniquely targeted to synaptic structures [305,306]. Critically, strong evidence also supports a fundamental relationship between microglia and the dopaminergic system. Based on the available scientific evidence, which is reviewed in detail below, we propose a (potentially) cyclic model highlighting the interrelationships between microglia, the DA system and synaptic function (Figure 4).

First, microglia and the DA system are highly interrelated, whereby microglia are highly prevalent in the basal ganglia nuclei (i.e., NAc, VTA, SN [307,308]) and express functional D1- and D2-like receptors [309,310,311], as well as DAT [312]. Functionally, prominent alterations in microglial morphology [312], enhanced microglial migration [309] and enhanced assembly of vimentin filaments [312] have been observed in “resting” microglia following DA treatment. Microglia are also involved in the wiring of the embryonic forebrain circuit, including dopaminergic axon outgrowth and positioning of neocortical interneurons, a process which is altered in cases of microglial dysfunction (i.e., via cell-depletion or genetic mutants [313]).

Second, microglia’s environmental surveillance is uniquely targeted to synaptic structures, whereby “resting” microglial processes localize with both pre- and post-synaptic structures, including dendritic spines [305,306]. During early brain development, microglia are involved in either the phagocytic [314,315] or trogocytotic [316] elimination of synapses, playing a critical role in synaptic pruning, a regressive event that is vital for neural circuit refinement and maturation. However, absence of either the fractalkine receptor (Cx3cr1 [314,317]) or complement receptor 3 (CR3 [315]) precludes synaptic pruning and results in immature synaptic connectivity. Microglia’s role in synaptic pruning continues through adolescence, whereby microglia transiently engulf dendritic spines in the PFC [318], and into adulthood [319].

In addition to synaptic pruning, microglia play a critical role in synaptic formation during development [316,317,320,321] and adult neurogenesis [322,323,324]. Specifically, microglia–dendrite interactions promote filopodia-like (i.e., immature postsynaptic protrusions that may develop into mature dendritic spines) formation [316,321]. Alterations in the gene expression of Cx3cr1, which lead to a depletion of microglia, however, precluded spine formation [317]. Furthermore, microglia regulate adult neurogenesis via multiple mechanisms, including phagocytosis [322] and the phagocytosis secretome [324], as well as via a nucleotide-mediated mechanism (i.e., ADP receptors P2Y12 and P2Y13 [323,324]) and the TAM family tyrosine kinases [324].

Notably, the dopaminergic system and synaptic structures may also interact with one another in a bidirectional manner. Dopaminergic afferents predominantly establish synaptic contact on the dendritic spine neck [325], and postsynaptic D1 and D2 receptors are localized in perisynaptic sites, supporting the anatomical interrelationship between the DA system and dendritic spines [326]. DA depletion results in prominent structural alterations in medium spiny neurons (MSNs) of the NAc, including decreased dendritic spine density [327,328] and decreased density of asymmetric synaptic contacts [329,330]. Additionally, a preferential loss of ‘thin’ spines, and a corresponding relative increase in ‘stubby’ spines, has also been reported in MSNs of the NAc following DA denervation [328]. Collectively, evidence supports a strong relationship between microglia, dopaminergic system function and synaptic function. To date, however, it is unknown whether the relationships between microglia and synaptic function or microglia and DA system function are bidirectional.

### 6.2. Disturbances of Brain Homeostasis: HIV-1

Early in the course of infection, HIV-1-infected monocytes migrate across the blood–brain barrier, infiltrating the brain and infecting microglia [331,332]. During HIV-1 infection, increased expression of microglial markers (e.g., CD68, MHC II) in the brain has often been interpreted as microglial activation [333], and more recent evidence supports morphological changes associated with microglial activation (i.e., amoeboid [334]). Additionally, HIV-1 infection likely leads to microglial dysfunction, as evidenced by cellular senescence [335]. Given the strong interrelationships between microglia, the DA system and synaptic function, microglial dysfunction may underlie the hypodopaminergic state (reviewed in Section 3) and synaptic dysfunction [195,336,337] commonly observed following chronic HIV-1 viral protein exposure (Figure 4B).

First, alterations in the relationship between microglia and DA system function have been observed following induction of the HIV-1 viral protein, Tat [338]. Specifically, Tat simultaneously decreased the number of microglia (i.e., Iba1 immunoreactive cells) and the number of dopamine neurons (i.e., tyrosine hydroxylase positive neurons) in the substantia nigra pars compacta, while an impact of HIV-1 Tat induction was not observed in the VTA [338]. Second, microglial activation and/or dysfunction may underlie HIV-1-associated synaptic dysfunction. HIV-1 viral proteins disrupt microglial proteins and receptors (e.g., Cx3cr1 [339], CR3 [340]) that underlie microglia-mediated neurite and pre- and post-synaptic engulfment [314,315,317]. Finally, chronic HIV-1 viral proteins may alter the bidirectional relationship between the dopaminergic system and synaptic structures. Specifically, in MSNs of the NAc, DA denervation induces a preferential loss of ‘thin’ spines, and a corresponding relative increase in ‘stubby’ spines [328], morphological changes which are consistent with the prominent shift towards ‘stubby’ spines reported following chronic HIV-1 viral protein exposure [337,341]. Thus, we posit that the activation and/or dysfunction of microglia underlies the prominent synaptic and dopaminergic system dysfunction observed in HIV in the post-cART era. Future studies directly investigating how these interrelationships are altered following chronic HIV-1 viral protein exposure have the potential to enhance our understanding of the neural mechanisms underlying HAND and identify novel targets for therapeutic development.

## 7. Conclusions

1. Dopamine values are dependent upon not only brain region, but also experimental approach (i.e., HPLC, microdialysis, or FSCV). Substantial variability in basal dopamine values may reflect differences in experimental parameters, and innovative genetic fluorescent probes may be a future direction for assessing dopamine signaling.
2. Results overwhelmingly support decreased dopamine concentrations following chronic HIV-1 viral protein exposure in either HIV-1 seropositive humans or biological systems utilized to model HIV-1. Therefore, future therapeutic approaches and models for the neurological complications of HIV-1 need to focus on rectifying decreased dopamine levels.
3. The clinical symptoms, including cognitive impairments and apathetic behaviors, reflect persistent dopamine deficits in HIV-1 seropositive individuals. There is no clinical evidence supporting increased dopamine following chronic HIV-1 infections.
4. To date, there is no compelling evidence that cART has any direct pharmacological action on the dopaminergic system—dopamine deficits persist in the current era of HIV-1 therapeutics.
5. HIV-1 infection likely leads to microglial dysfunction, which may have mechanistic implications for a chronic bidirectional interaction between low dopamine levels and synaptic dysfunction, implicated as neural mechanisms of HAND.

## Figures and Tables

**Table 1:** Estimated basal dopamine (DA) values. Abbreviations: High Performance Liquid Chromatogrphy (HPLC); Fast Scan Cyclic Voltammetry (FSCV).

Brain Region	Methodology	Estimated DA Concentration in ng/g of Tissue (X ± SEM)	Relative Standard Error	References
Amygdala	HPLC	3683.85 ± 3097	84.1%	[39,40,41,42,43]
	Microdialysis	0.06 ± 0.03	50%	[44,45,46,47]
Caudate	HPLC	16,365.9 ± 12,341.04	75.4%	[28,39,41,48,49,50,51,52,53,54]
	Microdialysis	0.88 ± 0.66	75%	[44,55,56]
Frontal Cortex	HPLC	200.73 ± 84.41	42.1%	[28,39,42,52,54,57,58,59,60,61,62]
	Microdialysis	0.23 ± 0.10	43.5%	[44,63,64,65]
Nucleus Accumbens	HPLC	35,772.90 ± 12,020.28	33.6%	[41,42,43,51,52,57,59,60,62,66,67,68]
	Microdialysis	0.76 ± 0.13	17.1%	[44,45,47,55,63,64,65,67,68,69,70,71,72,73,74,75,76,77,78,79,80,81,82,83,84,85,86,87,88,89,90,91,92,93,94,95,96,97,98,99]
	FSCV	6.95 ± 1.93	27.8%	[100,101,102,103,104]
Striatum	HPLC	67,460.52 ± 29,013.28	43%	[59,61,62,66,67,105,106,107,108,109,110,111]
	Microdialysis	1.42 ± 0.40	28.2%	[64,73,74,76,79,82,85,112,113,114]
Ventral Tegmental Area	HPLC	9200		[66]
	Microdialysis	0.25 ± 0.07	28%	[75,83]

**Table 2:** Influence of HIV-1 viral protein exposure on dopamine (DA) concentration relative to controls. Asterisks (*) indicate manuscripts that measured DA metabolites (e.g., homovanillic acid). Symbols: DA concentration is decreased (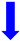) or increased (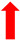) relative to controls. The equal sign (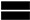) indicates no statistically significant differences in DA concentration between HIV and controls.

References	DA Concentration Relative to Controls	Virus	Brain Region	Species	Method
Larsson et al., 1991 [16]	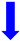	HIV	CSF	Human	HPLC *
Berger et al., 1994 [14]	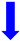	HIV	CSF	Human	HPLC
Sardar et al., 1996 [15]	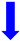	HIV	Caudate Nucleus	Human	HPLC
Di Rocco, 2000 [17]	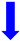	HIV	CSF	Human	HPLC *
Czub et al., 2001 [181]	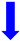	SIV	Hippocampus	Primate	HPLC
	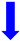		PFC		
	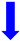		Putamen		
Koutsilieri, 2002 [182]	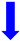	HIV	Striatum	Primate	HPLC
Jenuwein et al., 2004 [183]	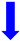	SIV	NAc	Primate	HPLC
Scheller et al., 2005 [184]	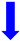	SIV	Putamen	Primate	HPLC
Kumar et al., 2009 [28]	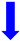	HIV	Caudate Nucleus	Human	HPLC
	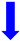		Globus Pallidus		
	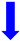		Putamen		
	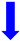		Substantia Nigra		
Ferris et al., 2009 [185]	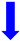	Tat Protein	Striatum	Rat	Microdialysis
Scheller et al., 2010 [174]	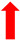	Early HIV	CSF	Human	HPLC
Kumar et al., 2011 [29]	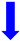	HIV	Caudate Nucleus	Human	HPLC
	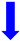		Globus Pallidus		
	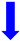		Putamen		
	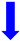		Substantia Nigra		
Kesby et al., 2016 [175]	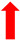	Acute Tat Protein	Caudate Putamen	Mouse	HPLC
	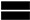		NAc		
	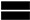	Tat Protein	Caudate Putamen	Mouse	HPLC
	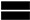		NAc		
Kesby et al., 2016 [178]	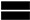	Acute Tat Protein	Caudate Putamen	Mouse	HPLC
	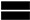		Hippocampus		
	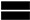		PFC		
	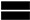		OFC		
Horn et al., 2017 [186]	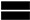	HIV	CSF	Human	HPLC
Javadi-Paydar et al., 2017 [187]	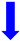	HIV-1 Proteins	NAc	Rat	Ex vivo slice voltammetry
Denton et al., 2019 [30]	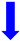	HIV-1 Proteins	NAc	Rat	FSCV
Saloner et al., 2020 [188]	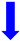	HIV	CSF	Human	HPLC
Strauss et al., 2020 [176]	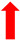	Acute Tat Protein	PFC	Mouse	HPLC
	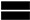		Striatum		
Denton et al., 2021 [189]	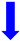	HIV-1 Proteins	NAc	Rat	FSCV

## Data Availability

All relevant data are within the manuscript.

## Supplementary Materials

The following are available online at https://www.mdpi.com/article/10.3390/cells10082158/s1, Table S1: Basal Dopamine (DA) values reported by individual manuscript.
