# Supplementary material for: HIV-Associated Apathy/Depression and Neurocognitive Impairments Reflect Persistent Dopamine Deficits
Source: Cells. 2021 Aug 21;10(8):2158. doi: 10.3390/cells10082158 (PMC8392364; doi:10.3390/cells10082158)
Supplement: Supplementary file 1 [file cells-10-02158-s001.zip › cells-1310001-supplementary.pdf]

Table S1. Basal Dopamine (DA) values reported by individual manuscript.

Reported DA Concentration	Average DA Concentration (ng/g; X ± SEM)	Species	Sex	Age	Reference
Amygdala, HPLC					
2.17 nmol/g	332.4	Primate	Male		Elchisak et al., 1983 [39]
1.88 ± 0.144 ng/mg	1880 ± 144	Rat	Male		Bradbury et al., 1985 [40]
0.11 ± 0.037 pmol/mg	16.85 ± 5.63	Human	Male & Female	57-82 Years	Ebinger et al., 1987 [41]
0.19 ± 0.03 ng/mg	190 ± 30	Rat	Male		Carvalho et al., 2005 [42]
16 ± 6.44 ng/mg	16000 ± 6440	Mouse	Male & Female	2-6 Months	Fadok et al., 2010 [43]
Amygdala, Microdialysis					
0.15 ± 0.02 fmol/μL	0.023 ± 0.003	Rat	Male		Inglis & Moghaddam, 1999 [44]
0.785 ± 0.257	0.122 ± 0.058	Rat	Male		Weiss et al., 2000 [45]
0.9 ± 0.45 pg/10 μL	0.09 ± 0.046	Rat	Male	4-5 Months	Tor-Agbidye et al., 2001 [46]
0.667 ± 0.31 pg/mL	0.0007 ± 0.0003	Rat	Male	9 Weeks	Adachi et al., 2013 [47]
Caudate, HPLC					
58.2 nmol/g	8915.1	Primate	Male		Elchisak et al., 1983 [39]
20.32 ± 7.66 pmol/mg	3113 ± 1773	Human	Male & Female	57-82 Years	Ebinger et al., 1987 [41]
10.9 ± 0.67 μg/g	10900 ± 670	Sheep	Female		Juorio & Chedrese, 1990 [48]
3.1 ± 0.8 ng/mg	3100 ± 800	Human	Male & Female	40.4 Years	Davis & Sparks, 1995 [49]
3.43 ± 0.22 ng/mg	3430 ± 220	Human	Male & Female	74 ± 2 Years	Wilson et al., 1996 [50]
1905 ± 195	1905 ± 195	Human	Male & Female	35-78 Years	Mushoff et al., 2000 [51]
127 ± 6.6 ng/mg	12700 ± 6600	Primate	Male & Female		Elsworth et al., 2008 [52]
4833 ± 531 ng/g	4833 ± 531	Human	Male & Female	80.6 Years	Rajput et al., 2008 [53]
3.9 ± 0.345	7.94 ± 16.553	Human	Male & Female	35-66 Years	Kumar et al., 2009 [28]
2969 ± 803 fmol/mg	455 ± 123	Human		74 ± 2 Years	Goldstein et al., 2011 [54]
Caudate, Microdialysis					
21.5 ± 0.4% fmol/min	2.19 ± 0.009%	Rat	Female	Adult	Robinson & Camp, 1990 [55]
1.68 ± 0.21 fmol/μL	0.257 ± 0.032	Rat	Male		Inglis & Moghaddam, 1999 [44]
1.7 ± 0.165 nM	0.18 ± 0.005	Mouse	Male	8-16 Weeks	Bosse & Mathews, 2011 [56]
Frontal Cortex, HPLC					
0.098 nmol/g	14.94	Primate	Male		Elchisak et al., 1983 [39]
0.053 ± 0.025 ug/g	52.5 ± 25	Rat	Male & Female	150 Days	Lucas & McMillen, 2002 [57]
0.08 ± 0.015 ng/mg	80 ± 15	Rat	Male		Carvalho et al., 2005 [42]
0.92 ± 0.09	920 ± 90	Primate	Male & Female		Elsworth et al., 2008 [52]
210.2 ± 14.8 fmol/mg	32.2 ± 2.27	Rat	Female	8-10 Weeks	Wisman et al., 2008 [58]
2.056 ± 0.166 log_{10} pg/g	0.118 ± 0.056	Human	Male & Female	35-66 Years	Kumar et al., 2009 [28]
83 ± 63 fmol/mg	12.71 ± 9.65	Human		74 ± 2 Years	Goldstein et al., 2011 [54]
215 ± 45 pg/mg	215 ± 45	Rat	Male		Bromek et al., 2011 [59]
0.475 ± 0.084 ng/mg	475 ± 84	Rat	Male	78 Days	Choi et al., 2012 [60]
113.83 ± 60.97 ng/g	113.83 ± 60.97	Rat	Male & Female		Hu et al., 2014 [61]
291.7 ± 55.4 pg/mg	291.7 ± 55.4	Rat	Male		Rysz et al., 2015 [62]
Frontal Cortex, Microdialysis					

2.7 ± 0.5 nM	0.41 ± 0.08	Rat	Male		Moghaddam & Bunney, 1989 [63]
2.55 ± 0.2 nM	0.391 ± 0.03	Rat	Female		Maisonneuve et al., 1991 [64]
0.23 ± 0.03 fmol/ µL	0.035 ± 0.005	Rat	Male		Inglis & Moghaddam, 1999 [44]
0.44 ± 0.09 pg/6 µL	0.074 ± 0.015	Rat	Female		Shoblock et al., 2003 [65]
Nucleus Accumbens, HPLC					
14.93 ± 3.393 pmol/mg	2287 ± 519.7	Human	Male & Female	57-82 Years	Ebinger et al., 1987 [41]
622 ± 97 ng/g	622 ± 97	Human	Male & Female	35-78 Years	Mushoff et al., 2000 [51]
48.5 ± 1.55 µg/g	4850 ± 1550	Rat	Male & Female	150 Days	Lucas & McMillen, 2002 [57]
10.8 ± 0.9 ng/mg	10800 ± 900	Rat	Male		Carvalho et al., 2005 [42]
75.7 ± 6 ng/mg	75700 ± 6000	Primate	Male & Female		Elsworth et al., 2008 [52]
71.5 ± 4.64 ng/mg	71500 ± 4640	Mouse	Male & Female	2-6 Months	Fadok et al., 2010 [43]
8700 ± 50 ng/g	8700 ± 50	Rat	Male		Bromek et al., 2011 [59]
45.51 ± 6.13 ng/mg	45510 ± 6130	Rat	Male	78 Days	Choi et al., 2012 [60]
75 ± 4 ng/mg	75000 ± 4000	Rat			Salvatore et al., 2012 [66]
4535.8 ± 251.4 pg/mg	4535.8 ± 251.4	Rat	Male	3-28 Months	Rysz et al., 2015 [62]
125 ± 5 ng/mg	125000 ± 25000	Mouse		12-15 Weeks	Winner et al., 2017 [67]
4.77 ± 1.15 ng/mg	4770 ± 1150	Mouse	Male		Bergamini et al., 2018 [68]
Nucleus Accumbens, Microdialysis					
8 ± 1 pg/20 µL	0.4 ± 0.051	Rat	Male		Hernandez & Hoebel, 1988 [69]
17.4 ± 1.2 nM	2.67 ± 0.18	Rat	Male		Moghaddam & Bunney, 1989 [63]
9.838 ± 2.828 nM	1.505 ± 0.432	Rat	Male		Pettit et al., 1990 [70]
15 ± 0.4% fmol/min	1.53 ± 0.006%	Rat	Female	Adult	Robinson & Camp, 1990 [55]
0.01 ± 0.001 pmol/20 µL	0.08 ± 0.008	Rat	Male	Adult	Pothos et al., 1991 [71]
14.27 ± 1.1 nM	2.19 ± 0.17	Rat	Female		Maisonneuve et al., 1991 [64]
4.05 ± 0.2 nM	0.62 ± 0.03	Rat	Male		Parsons & Justice, 1992 [72]
10.02 ± 1.45 nM	1.53 ± 0.22	Rat	Female		Maisonneuve & Glick, 1992 [73]
0.171 ± 0.085 pmol/20 µL	0.655 ± 0.326	Rat	Female		Chapman et al., 1992 [74]
58.7 ± 6.9 fmol/40 µL	0.23 ± 0.026	Rat	Male		Chen et al., 1993 [75]
10.855 ± 1.675 nM	1.665 ± 0.255	Rat	Male & Female	90-120Days	Blanchard et al., 1993 [76]
0.14 ± 0.005 pmol/25 µL	0.86 ± 0.012	Rat	Male		Anagnostakis & Spyraki, 1994 [77]
3.275 ± 0.408 nM	0.5 ± 0.0615	Rat	Male	90-150 Days	Hemby et al., 1995 [78]
0.0375 ± 0.003 pmol/ 15 µL	0.379 ± 0.025	Rat	Male & Female	Adults	Blanchard & Glick, 1995 [79]
32 ± 4 fmol/20 µL	1.2 ± 0.15	Rat	Female		Campbell & McBride, 1995 [80]
3.45 ± 0.64 nM	0.528 ± 0.098	Rat	Male		Weiss et al., 1996 [81]
0.0197 ± 0.003 pmol/20 µL	0.151 ± 0.023	Rat	Female		Pearl et al., 1996 [82]
73 ± 16 fmol/20 µL	0.559 ± 0.1	Rat	Female	Adult	Kohl et al., 1998 [83]

1.428 ± 0.208 fmol/µL	0.219 ± 0.032	Rat	Male		Yan, 1999 [84]
1.24 ± 0.44 fmol/µL	0.189 ± 0.067	Rat	Male		Inglis & Moghaddam, 1999 [44]
0.01 ± 0.002 pmol/10 µL	0.15 ± 0.03	Rat	Female		Maisonneuve & Glick, 1999 [85]
2.375 ± 0.685 nM	0.364 ± 0.105	Rat	Male		Weiss et al., 2000 [45]
8.8 ± 0.4 nM	1.3 ± 0.06	Rat	Male		Yim & Gonzales, 2000 [86]
0.006 ± 0.003 pmol/10 µL	0.09 ± 0.05	Rat	Female		Szumlinski et al., 2000 [87]
0.015 ± 0 pmol/10 µL	0.23 ± 0	Rat	Female		Szumlinski et al., 2000 [88]
10.3 ± 1.8 nM	1.58 ± 0.28	Rat	Female		Johnson et al., 2000 [89]
66.8 ± 3.3 fmol/50 µL	0.2 ± 0.01	Rat	Male		Fadda et al., 2003 [90]
0.014 ± 0.003 pmol/10 µL	0.22 ± 0.047	Rat	Female		Steinmiller et al., 2003 [91]
2.46 ± 0.39 pg/10 µL	0.25 ± 0.04	Rat	Female		Shoblock et al., 2003 [65]
1.565 ± 0.22 pg/20 µL	0.078 ± 0.011	Rat	Male & Female	10 Days	Kosten et al., 2003 [92]
4.267 ± 1.867 nM	1.557 ± 0.287	Rat	Male	90-150 Days	Smith et al., 2006 [93]
0.023 ± 0.002 pmol/25 µL	0.14 ± 0.012	Rat	Female	3 Months	Geiger et al., 2009 [94]
1.14 ± 0.14 nM	0.175 ± 0.021	Rat	Male		Borgkvist et al., 2012 [95]
0.02 ± 0.004 pmol/10 µL	0.31 ± 0.06	Rat	Female		McCallum et al., 2012 [96]
0.59 ± 0.163 pg/ µL	0.593 ± 0.157	Rat	Male	9 Weeks	Adachi et al., 2013 [47]
4.18 ± 1.025 pg/40 µL	0.105 ± 0.026	Rat	Male & Female	Early Adolescence	Grotewold et al., 2014 [97]
6.445 ± 1.245 pg/10 min	0.43 ± 0.083	Rat	Male & Female	50-55 Days	Cummings et al., 2014 [98]
0.022 ± 0.001 pmol/ µL	3.31 ± 0.18	Rat	Female		Eggan & McCallum, 2016 [99]
Nucleus Accumbens, FSCV					
20.35 ± 0.265 nM	3.12 ± 0.04	Rat	Male		Roitman et al., 2008 [100]
20-30 nM	3.1 – 4.6	Rat	Male		Owesson-White et al., 2012 [101]
~49 nM	7.5	Rat	Male		Dreyer et al., 2016 [102]
90 ± 9 nM	14 ± 1.4	Mouse			Atcherley et al., 2015 [103]
41 ± 13 nM	6.3 ± 2	Rat	Male		Johnson et al., 2018 [104]
Striatum, HPLC					
0.64 ± 0.05 nmol/mg	98035 ± 7659	Mouse	Male	7 Weeks	Kita et al., 2000 [105]
11500 ± 1500 pg/mg	11500 ± 1500	Mouse	Male	9-19 Weeks	Bałkowiec-Iskra et al., 2007 [106]
29.175 ± 21 ng/mg	279175 ± 21000	Mouse	Male	9-16 Weeks	Petzinger et al., 2007 [107]
11.3 ± 1.562 nmol/g	1731 ± 239.384	Rat	Female	2-3 Months	Swiercz et al., 2009 [108]
9800 ± 130 pg/mg	9800 ± 130	Rat	Male		Bromek et al., 2011 [59]
165 ± 13 ng/mg	165000 ± 13000	Rat			Salvatore et al., 2012 [66]
1901.4 ± 64.78 ng/g	1901.4 ± 64.78	Rat	Male & Female		Hu et al., 2014 [61]
5 ± 0.6 ng/mg	5000 ± 60	Rat	Male	3-20.5 Months	Villar-Cheda et al., 2014 [109]
6553.8 ± 331.6 ng/g	6553.8 ± 331.6	Rat	Male		Rysz et al., 2015 [62]
1280 ± 90 ng/g	1280 ± 90	Rat	Male		Nikishina et al., 2016 [110]

225 ± 30 ng/mg	225000 ± 30000	Mouse		3-28 Months	Winner et al., 2017 [67]
4.55 ± 0.45 ng/mg	4550 ± 450	Rat and Mouse	Male	3.5-4.5 Months	Garrido-Gil et al., 2018 [111]
Striatum, Microdialysis					
24 ± 1.6 nM	3.676 ± 0.25	Rat	Female		Maisonneuve et al., 1991 [64]
14.49 ± 1.9 nM	2.22 ± 0.29	Rat	Female		Maisonneuve & Glick, 1992 [73]
0.105 ± 0.025 pmol/20 min	0.402 ± 0.096	Rat	Female		Chapman et al., 1992 [74]
17.435 ± 2.81 nM	2.671 ± 0.43	Rat	Male & Female	90-120 Days	Blanchard et al., 1993 [76]
0.052 ± 0.006 pmol/15 μL	0.521 ± 0.061	Rat	Male & Female	Adult	Blanchard & Glick, 1995 [79]
0.026 ± 0.007 pmol/20 μL	0.196 ± 0.054	Rat	Female		Pearl et al., 1996 [82]
0.026 ± 0.003 pmol/10 μL	0.4 ± 0.038	Rat	Female		Maisonneuve & Glick, 1999 [85]
5 fmol/ μL	0.77	Primate	Male		Bradberry, 2000 [112]
18 ± 3 nM	2.76 ± 0.46	Rat	Male		Shou et al., 2006 [113]
4 nM	0.61	Mouse	Male	4 or 10 Weeks	Zhang et al., 2009 [114]
Ventral Tegmental Area, HPLC					
9.2 ± 1.4 ng/mg	9200 ± 1400	Rat			Salvatore et al., 2012 [66]
Ventral Tegmental Area, Microdialysis					
46.5 ± 3.9 fmol/40 μL	0.178 ± 0.015	Rat	Male		Chen et al., 1993 [75]
42 ± 8 fmol/20 μL	0.322 ± 0.061	Rat	Female	Adult	Kohl et al., 1998 [83]
